# Death of Monocytes through Oxidative Burst of Macrophages and Neutrophils: Killing in Trans

**DOI:** 10.1371/journal.pone.0170347

**Published:** 2017-01-18

**Authors:** Viviane Ponath, Bernd Kaina

**Affiliations:** Department of Toxicology, University Medical Center, Mainz, Germany; The University of Hong Kong, HONG KONG

## Abstract

Monocytes and their descendants, macrophages, play a key role in the defence against pathogens. They also contribute to the pathogenesis of inflammatory diseases. Therefore, a mechanism maintaining a balance in the monocyte/macrophage population must be postulated. Our previous studies have shown that monocytes are impaired in DNA repair, rendering them vulnerable to genotoxic stress while monocyte-derived macrophages are DNA repair competent and genotoxic stress-resistant. Based on these findings, we hypothesized that monocytes can be selectively killed by reactive oxygen species (ROS) produced by activated macrophages. We also wished to know whether monocytes and macrophages are protected against their own ROS produced following activation. To this end, we studied the effect of the ROS burst on DNA integrity, cell death and differentiation potential of monocytes. We show that monocytes, but not macrophages, stimulated for ROS production by phorbol-12-myristate-13-acetate (PMA) undergo apoptosis, despite similar levels of initial DNA damage. Following co-cultivation with ROS producing macrophages, monocytes displayed oxidative DNA damage, accumulating DNA single-strand breaks and a high incidence of apoptosis, reducing their ability to give rise to new macrophages. Killing of monocytes by activated macrophages, termed *killing in trans*, was abolished by ROS scavenging and was also observed in monocytes co-cultivated with ROS producing activated granulocytes. The data revealed that monocytes, which are impaired in the repair of oxidised DNA lesions, are vulnerable to their own ROS and ROS produced by macrophages and granulocytes and support the hypothesis that this is a mechanism regulating the amount of monocytes and macrophages in a ROS-enriched inflammatory environment.

## Introduction

Monocytes and macrophages are mononuclear phagocytes that play a crucial role in tissue homeostasis and immunity. Monocytes originate from myeloid precursor cells in primary lymphoid organs, including foetal liver and bone marrow, during embryonic and adult haematopoiesis [1]. Monocytes move via the bloodstream to peripheral tissues where they differentiate, depending on the local growth factors, cytokines, and microbial molecules, into macrophages or myeloid dendritic cells (DCs) [2]. Monocytes are also recruited to sites of infection and mediate anti-microbial activity against viruses, bacteria, fungi and protozoa [3,4]. As first line defence, monocytes and macrophages can phagocytose pathogens and foreign particles, release cytokines and generate reactive oxygen species (ROS) in a “respiratory burst” [5–7]. Monocytes and macrophages also contribute to the pathogenesis of inflammatory and degenerative diseases, *e*.*g*. atherosclerosis, adipose tissue inflammation and insulin resistance [1,8]. Oxidative stress at the site of inflammation promotes endothelial dysfunction and tissue injury resulting in migration of leukocytes across the endothelial barrier [9]. Since polymorphonuclear neutrophils and macrophages are the main ROS producers in the inflamed tissue, both after acute and chronic infection and tissue damage [9–11], it is reasonable to posit that they are quantitatively tightly regulated. How this occurs is a subject of intensive research.

Previously, we have shown that monocytes compared to macrophages and DCs are hypersensitive towards DNA damaging insults such as alkylating agents (MNNG and temozolomide), ionising radiation and oxidising agents (oxidised low density lipoproteins, *tert*-butyl hydroperoxide and hydrogen peroxide) [12,13]. This is the result of a DNA repair defect in monocytes. Thus, the DNA repair pathways for base excision repair (BER) and DNA double-strand break (DSB) repair are down-regulated in monocytes as they lack the DNA repair proteins XRCC1, ligase IIIα, ligase I, poly(ADP-ribose) polymerase-1 (PARP1) and the catalytic subunit of DNA-dependent protein kinase (DNA-PK_cs_) [12,13]. We also showed that the expression of these repair proteins is controlled by cytokines since during maturation of monocytes into macrophages triggered by GM-CSF the DNA repair proteins become up-regulated and, therefore, macrophages become repair-proficient and resistant to exogenous oxidative agents, ionising radiation and chemical genotoxicants including anticancer drugs. The same occurs when monocytes are stimulated by IL-4 and GM-CSF for maturation into dendritic cells, which then become resistant to genotoxic agents [12,13].

Based on the finding that XRCC1 and other DNA repair factors are downregulated in monocytes, we proposed the hypothesis that the DNA repair defect in monocytes is of biological relevance as it may regulate the amount of monocytes and macrophages in a ROS highly enriched environment, *i*.*e*. in the inflamed tissue [13]. In this study we set out to provide support for this hypothesis by demonstrating that the ROS burst of monocytes and activated macrophages in their vicinity is strong enough to kill monocytes, which was anticipated because of their DNA repair defect. To this end, we first studied the effect of a ROS burst on the survival of monocytes freshly isolated from peripheral blood of healthy donors and macrophages derived from them by GM-CSF. Further, we assessed in co-culture experiments the effect of ROS produced by macrophages on monocyte survival, and extended these studies by co-culture experiments with activated neutrophilic granulocytes. The data revealed that monocytes are sensitive to ROS they produce following activation while macrophages are protected, although similar amounts of initial oxidative DNA lesions were induced. They also revealed that monocytes co-cultured with ROS-producing macrophages showed increased oxidative DNA damage (8-oxo-guanine) levels, DNA breaks and cell death and were also impaired in generating new macrophages. The data support the hypothesis that the DNA repair defect in monocytes has a biological function, namely it is involved in a regulatory feedback loop in the innate immune response. During this negative feedback loop, monocytes are killed by the ROS burst generated by activated macrophages and neutrophils in their vicinity, termed *killing in trans*, which prevents from the generation of excessive numbers of new macrophages and thereby reduce the overall ROS level in the inflamed tissue.

## Materials and Methods

Study was performed according to the rules of the ethic commission of the University.

### Chemicals and reagents

Phorbol 12-myristate 13-acetate (PMA; InvivoGen, Toulouse, France) stock solution was prepared at 500 ng/μl in DMSO and stored at -20°C. For experiments 50 ng/μl solutions were freshly prepared in PBS. ROS dye CM-H2DCFDA (Life Technologies GmbH, Darmstadt, Germany) was prepared fresh in DMSO to a stock concentration of 1.25 mM. 250 μM luminol (Sigma-Aldrich, Munich, Germany) stock solutions were prepared in DMSO and stored at -20°C. 5 mM stock solutions of 5(6)-carboxyfluorescein diacetate *N*-succinimidyl ester (CFSE; Fluka/Sigma-Aldrich, Germany) were stored at -20°C. *N*,*N'*-dimethylthiourea (DMTU) was freshly prepared for each experiment (Sigma-Aldrich). 2′(3′)-O-(4-Benzoylbenzoyl) ATP (bzATP, Sigma-Aldrich) and lipopolysaccharide (LPS, *E*.*coli* 0127:B8, Sigma-Aldrich) were stored at -20°C. CD14 Vioblue (TÜK4), CD15 APC (VIMC6) and Annexin V FITC were purchased from Miltenyi Biotec (Bergisch Gladbach, Germany). Annexin V APC was purchased from BioLegend/BIOZOL (Munich, Germany). 10H antibody was a kind gift from Prof. Alexander Bürkle (University of Konstanz, Germany). 0.8 x 10^6^ U /ml granulocyte macrophage colony-stimulating factor (GM-CSF, Sanofi Aventis, USA) stock solution was prepared in X-Vivo 15 and stored at -20°C. Formamidopyrimidine DNA glycosylase (FPG) was a kind gift of Prof. Bernd Epe (University of Mainz, Germany). Horse radish peroxidase was a kind gift of Dr. Jörg Fahrer.

### Generation of monocytes, T cells, granulocytes and macrophages from peripheral blood mononuclear cells (PBMCs)

For the isolation of monocytes and subsequent differentiation into macrophages, peripheral blood mononuclear cells (PBMCs) were isolated by Ficoll-Hypaque density gradient centrifugation from buffy coats of blood donors obtained from the blood bank of the University Medical Center Mainz. PBMCs were washed four times in PBS + 2 mM EDTA + 0.5% BSA and then seeded at 1.5 x 10^7^ cells / 3 ml / well in six-well plates (Corning/Costar, Bodenheim, Germany) in RPMI 1640 (Gibco/Life Technologies, Darmstadt, Germany) + 1.5% heat-inactivated autologous plasma for 30 min at 37°C and 5% CO_2_. Non-adherent cells (T cells) were gently washed away with PBS. Adherent cells represented monocytes, which were incubated in RPMI 1640 + 10% heat-inactivated autologous plasma for at least two hours to detach from the plate again. Monocytes were harvested and cultured in X-Vivo 15 (Lonza, Cologne, Germany). For the generation of macrophages, monocytes were seeded at 0.7 x 10^6^ cells / well / ml in 24 well plates in X-Vivo 15 + 800 U / ml GM-CSF and harvested on day six. Only adherent cells were considered macrophages and used for experiments.

Granulocytes were isolated by the Optiprep density method. First, two Optiprep solutions of different densities (1.077 g/ml and 1.095 g/ml) were prepared from Optiprep (density 1.32 g / ml, ProgenBiotechnik GmbH, Heidelberg, Germany) diluted in 0.85% NaCl, 1 mM EDTA, 20 mM HEPES pH 7.4. Then, 9 ml blood from buffy coats was mixed with 1 ml 6% dextran sulfate sodium salt (Sigma-Aldrich) dissolved in 0.85% NaCl buffer and incubated for 1 h at RT until a clear upper phase became visible. The upper phase was transferred into a fresh 15 ml tube containing an upper layer of 4 ml Optiprep (1.077 g/ml) and a lower layer of 4 ml Optiprep (1.095 g/ml). The tubes were centrifuged at 1900 rpm for 30 min without brake. Two rings of cells became visible and the lower one was collected. The cells were washed in PBS + 2 mM EDTA + 0.5% BSA and then seeded in RPMI 1640 + 1.5% heat-inactivated plasma for 30 min at 37°C. Non-adherent cells were removed and adherent granulocytes were allowed to detach in the presence of RPMI 1640 + 10% heat-inactivated plasma for 2 h. Afterwards granulocytes were seeded at 1 x 10^6^ cells/ml in X-Vivo 15 medium. For purity testing, neutrophils were stained with CD15 antibody and checked via flow cytometry or immunofluorescence (see supportive information S1 Fig).

### Detection of extracellular and intracellular ROS

For intracellular ROS production, 1 x 10^6^ cells per ml X-Vivo 15 were loaded with 10 μM CM-H2DCFDA (Life Technologies, Darmstadt, Germany). Immediately after loading, cells were treated with 100 ng/ml PMA or 100 μM *tert*-butyl hydroperoxide (t-BOOH) for 30 min at 37°C and 5% CO_2_. Macrophages were detached from the plate by adding 500 μl accutase (PAA, Pasching, Austria) for 20 min at 37°C. Both monocytes and macrophages were washed with PBS, pelleted by centrifugation and resuspended in PBS before they were analysed by flow cytometry. Mean fluorescence intensity was determined with the FACS Canto II Diva software (Becton–Dickinson, Heidelberg, Germany). The detection of extracellular ROS has been described elsewhere [14]. Briefly, 0.5 x 10^6^ cells were suspended in 500 μl complete PBS (PBS with 0.5 mM MgCl_2_, 0.7 mM CaCl_2_ and 0.1% glucose). 10 μg/ml horseradish peroxidase and 10 μM luminol were added to the cells before activation with 100 ng/ml PMA. The chemiluminescence was measured in a Berthold TriStar^2^ LB 942 (Bad Wildbad, Germany) at RT for the indicated time. The area under the curve (AUC) was determined for quantification.

### Co-culture of monocytes with macrophages or granulocytes

Monocyte-derived macrophages were generated in 24-well plates. On day six, non-adherent cells were washed away and adherent macrophages were incubated in the presence of CFSE (5 μM for 8 min in the dark at RT) for labelling. Thereafter, the cells were washed twice in PBS + 0.5% BSA and once in PBS before 500 μl X-Vivo 15 was added. Cells were equilibrated for at least one hour at 37°C before they were used for co-culturing. Granulocytes were isolated on the same day as monocytes. They were labelled with CFSE similar to the macrophages, seeded at 1 x 10^6^ cells /ml in X-Vivo 15 and equilibrated for at least one hour at 37°C before the experiment.

Monocytes were freshly isolated on day six and resuspended at 1 x 10^6^ cells / ml in X-Vivo 15. For co-culture, 0.5 x 10^6^ monocytes were added to macrophages (approximately 1:1 ratio in 1 ml assay) before treatment. For PMA-pulse treatment, macrophages were treated with 100 ng/ml PMA for 15 min at 37°C; then the medium was removed, cells were washed with PBS and monocytes were added immediately afterwards. Granulocytes were treated for 10 min, then they were washed with PBS and re-suspended at 1 x 10^6^ cells/ml with X-Vivo 15 before monocytes were added. Chronic PMA treatment means that both cell types were exposed to PMA for the period of the assay. DMSO was used as solvent control. After treatment, monocytes and macrophages or monocytes and granulocytes were carefully removed from the plates by accutase (15 min, 37°C), stained with CD14 marker and subjected to flow cytometry. Macrophages and granulocytes were distinguished from monocytes by their CFSE signal, with which they were pre-labelled. For Annexin V staining see below. For co-culture experiments with LPS/bzATP, monocytes and macrophages were primed with 1 μg/ml LPS for 16 h before macrophages were pulsed with 250 μM bzATP for 15 min. The medium was removed, macrophages were washed with PBS and monocytes were added. Monocytes were stained for CD14 and exclusion of CFSE-positive macrophages. Cell death was measured 24 h after treatment.

### Quantification of cell death

Cell death was measured 24 or 48 h after treatment using Annexin V, APC or Annexin V and FITC. Monocytes were stained for CD14 and exclusion of CFSE-positive macrophages or granulocytes. Cells were resuspended in 50 μl Annexin V binding buffer (10 mM HEPES; pH 7.4, 140 mM NaCl, 2.5 mM CaCl_2_, 0.1% BSA) and Annexin V (2.5 μl per 1 x 10^6^ cells) and incubated on ice in the dark for 20 min before samples were topped up with 300 μl Annexin V binding buffer. Cell death was measured in a FACS Canto II (Becton–Dickinson, Heidelberg, Germany) and data was analysed using the FACS Canto II Diva software (for exemplary gating see S2 Fig). Life cell images of monocyte-derived macrophages were taken with an Olympus IX70 microscope and Olympus cell^F software.

### Single-cell gel electrophoresis (Comet Assay)

Oxidative DNA damage was measured by the formamidopyrimidine DNA glycosylase (FPG)-modified alkaline Comet Assay. Briefly, monocytes were co-cultured with PMA-activated macrophages or co-treated with 100 ng/ml PMA for 1 h. Monocytes were harvested, embedded in 0.5% low melting point agarose and transferred onto agarose-precoated slides. The slides were incubated in lysis buffer (2.5 M NaCl, 100 mM EDTA, 10 mM Tris, 10% DMSO, 1% Triton X, pH 10) for 1 h at 4°C. Slides were equilibrated 2 x 5 min in buffer F (40 mM HEPES, 0.1 M KCl, 0.5 mM EDTA, 0.2% BSA, pH 8.0) at RT. FPG was diluted in buffer F and 50 μl was added to the slides and incubated in a humid chamber at 37°C for 45 min. DNA unwound in electrophoresis buffer (300 mM NaOH, 1 mM EDTA, pH 13) for 22 min at 4°C before electrophoresis was performed for 20 min at 0.74 V / cm and 300 mA. Slides were washed three times in neutralisation buffer (0.4 M Tris, pH 7.5). The samples were fixed in 100% ethanol for 10 min at RT, air-dried and stained with 50 μg/ml propidium iodide. Comets were analysed by fluorescence microscopy using an Olympus BX50 equipped with a ColorView camera (Olympus, Münster, Germany). At least 100 cells were scored in each experiment by means of Comet IV software (Perceptive Instruments Ltd., Bury St Edmunds, UK).

### Immunofluorescence of 8-oxo-guanine, XRCC1, pATM, 53BP1 and poly(ADP-ribose)

Immunostaining of 8-oxo-guanine (8OHdG): For pulse treatment, macrophages were pulse-activated with 100 ng/ml PMA for 15 min. Then, the medium was removed, cells were washed with PBS and monocytes were added and co-cultured for 45 min. In parallel, both monocytes and macrophages were treated with PMA. After 1 h, monocytes in suspension were carefully removed from the adherent macrophages, centrifuged at 1500 rpm for 6 min at 4°C and the pellet resuspended in a small volume (< 5 μl) PBS. The monocytes settled on Superfrost slides (Gerhard Menzel GmbH/ThermoFisher, Braunschweig, Germany) and dried for approximately 5 min before fixation with -20°C 100% methanol for 15 min. Cells were washed twice in PBS and slides were transferred into a humidified chamber. RNA was digested with RNase A (200 μg / ml) and RNase T (50 U / ml) in PBS for 1 h at 37°C. Cells were washed once in PBS before the DNA was denatured in alkaline solution (70 mM NaOH, 140 mM NaCl, 40% (v/v) methanol) at 0°C for 5 min. Slides were washed 3 x 5 min in PBS. Protein was digested with pepsin (60 μg / ml in PBS-HCl pH 6.0) for 10 min at 37°C. Slides were washed in PBS before proteinase-K (2 μg/ml proteinase K, 20 mM Tris/HCl, 2 mM CaCl_2_, pH 7.5) digestion for 10 min at 37°C. Slides were washed with 0.2% glycine in PBS for 10 min. Samples were blocked in 0.5% casein in PBS + 0.1% Tween20 (PBS-T) for 1 h at RT. Primary mouse 8OHdG antibody (clone 15A3, Abcam, Cambridge, UK) was diluted 1:1000 in blocking solution and incubated over night at 4°C. Samples were washed 5 x 5 min in PBS-T. Secondary goat α-mouse antibody (clone: pAb-Cy3, Dianova, Hamburg, Germany) was diluted 1:600 in blocking solution and samples incubated for 1 h at RT. Afterwards, slides were washed 5 x 5 min in PBS-T and twice in Tris-NaCl buffer (0.1 M Tris-HCl, 2 mM MgCl_2_, 1 M NaCl, pH 7.5, 0.05% Triton X-100) for 15 min. DNA was stained with 100 μM ToPro3 (Invitrogen) for 15 min at RT. Samples were rinsed with PBS, mounted with one drop of Vectashield (Vector Labs, Burlingame, CA, USA) and cover slips fixed with nail polish. Images were acquired using a laser scanning microscope (LSM 710) and analysed using ZEN Software from Carl Zeiss and ImageJ.

Immunostaining of XRCC1, pATM and 53BP1: For XRCC1 the cells were seeded on cover slips in 6 cm petri dishes in the presence of GM-CSF and incubated for the indicated time points. For all stainings the cells were fixed in ice cold methanol:acetone (7:3 ratio) for 6 min at -20°C. The samples were rinsed with PBS and fixed with 2.5% paraformaldehyde for 10 min at RT. Samples were washed in PBS for 10 min and then blocked in 10% normal goat serum (Invitrogen, Life Technologies, Carlsbad, USA) for 1 h at RT. Primary XRCC1 antibody (ab134056, Abcam, Cambridge, UK), pATM (phospho 1981, ab81292, Abcam) and 53BP1 (A300-272A, Bethyl Laboratories, Inc) were diluted 1:1000 in 1% BSA in PBS-T and incubated overnight at 4°C. Samples were washed 3 x 5 min in PBS and incubated with secondary goat rabbit antibody (A11070 Alexa Fluor488, Invitrogen) for XRCC1 and pATM, and mouse antibody (A11017 Alexa Fluor 488, Invitrogen) for 53BP1 diluted 1:300 and 1:1400, respectively in 1% BSA in PBS-T for 1 h at RT. Afterwards, cover slips were washed 3 x 5 min in PBS and the DNA was stained with 100 μM ToPro3 for 15 min. Samples were rinsed with PBS, mounted with one drop of Vectashield cover slips were fixed onto glass slides with nail polish.

Immunostaining for poly(ADP-ribose) (PAR): Cells were seeded onto cover slips in 6 cm petri dishes. Cells were treated with 1 mM H_2_O_2_ for 5 min before they were fixed in ice cold methanol for 7 min at -20°C [15]. Cover slips were washed 3 x 5 min in PBS and samples were blocked in 5% dry milk in PBS-T for 1 h at RT. Primary PAR antibody (10H) was diluted 1:300 in blocking solution and incubated overnight at 4°C. Samples were washed 3 x 10 min and incubated with secondary goat mouse antibody (pAb-Cy3, Dianova) diluted 1:500 in blocking solution for 1 h at RT. Cover slips were washed 3 x 10 min in PBS and then DNA was stained using ToPro3 as described above. Cover slips were mounted onto glass slides and sealed with nail polish.

### Preparation of whole-cell extracts for western blot analysis

Cells were harvested, and lysed on ice in an appropriate amount of lysis buffer containing 25 mM Tris-HCl (pH 8.0), 500 mM NaCl, 1 mM EDTA, 1 mM PMSF, 2 mM DTT, 1 mM sodium orthovanadate, 0.5% NP-40 and 1x protease inhibitor (cOmplete, Mini, Roche). Samples were sonified for 2 x 10 pulses on ice and then centrifuged at 13.000 × g at 4°C for 15 min. The supernatant was recovered, boiled in loading buffer for 5 min at 95°C and 50 μg cell extract was separated on a 10% SDS polyacrylamide gel and blotted onto a nitrocellulose membrane for 2 h at 300 mA. Membranes were blocked with 5% BSA-PBS for 1 h at RT. The antibodies used were pChk2 (Thr68, 2661, Cell Signaling), CHK2 (2662 Cell Signaling), p53 (sc-81168, Santa Cruz), p-p53 (Ser46, 2521, Cell Signaling), GAPDH (sc-32233, Santa Cruz) and ERK2 (Santa Cruz).

### Statistical analysis

Results from at least three independent experiments are displayed as n ± standard error of mean (SEM). Statistical analysis of data was performed with GraphPad Prism5 Software.

## Results

**Fig 1:**
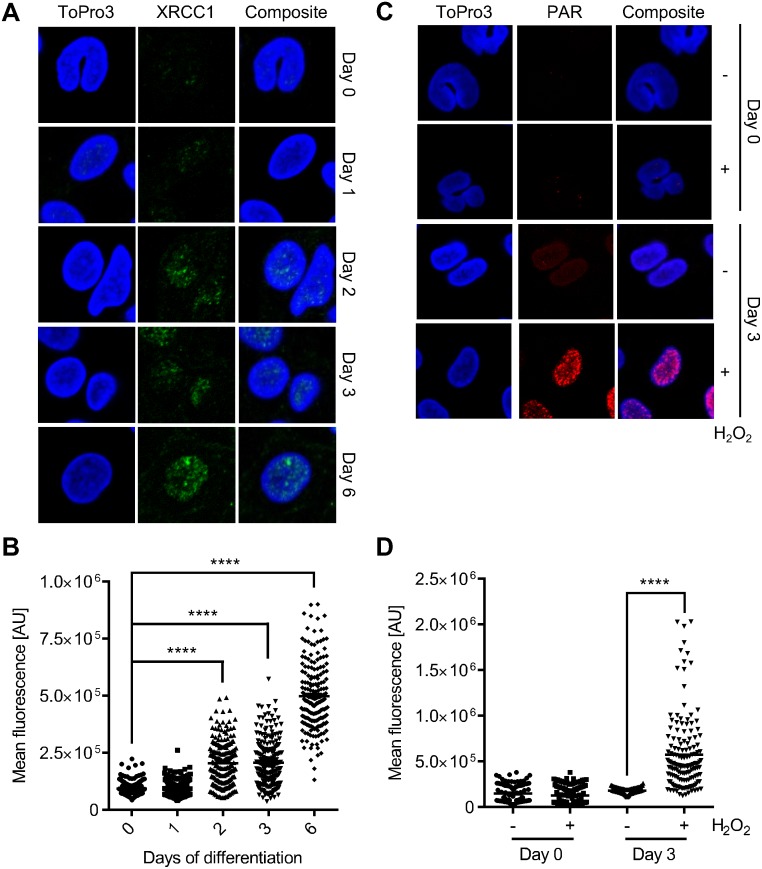
XRCC1 expression during macrophage maturation and poly(ADP-ribose) formation after hydrogen peroxide treatment in monocytes and monocyte-derived macrophages. (A) Representative images of immunofluorescence staining of XRCC1 in monocytes differentiating into macrophages through GM-CSF treatment over a period of 6 days. (B) Quantification of the mean fluorescence signal of XRCC1. Each dot represents the fluorescence intensity of a single cell. (C) Representative images of the immunofluorescence staining of PAR in monocytes and monocytes that were differentiated into macrophages (at day 3). The control displayed no PAR signal whereas cells treated with 1 mM H_2_O_2_ for 5 min showed increased levels of PAR in macrophages on day 3 of differentiation. (D) Quantification of the mean fluorescence signal of PAR. Each dot represents the fluorescence intensity of a single cell. Data are from two independent experiments with at least 50 cells counted for each sample ± SEM, 1-way ANOVA, Tukey’s Multiple Comparison Test, ****p < 0.0001.

Previously, we have shown that monocytes do not express detectable amounts of XRCC1 and PARP1, both key proteins in the BER and the alternative (back-up) non-homologous end joining (B-NHEJ) pathway [13,16]. Here, we substantiated this finding by means of immunocytochemistry, showing that monocytes are almost negative for nuclear XRCC1. However, during the differentiation of monocytes into macrophages, triggered by GM-CSF, nuclear XRCC1 levels increased significantly (Fig 1A for representative images and Fig 1B for quantification of the XRCC1 signal). The same was shown for poly(ADP)ribose (PAR), the product of PARP1 enzyme activity. It was barely detectable in monocytes and became clearly detectable already 3 days after the onset of GM-CSF treatment (Fig 1C for representative images and Fig 1D for quantification). The data confirm the impaired repair status of monocytes [12].

**Fig 2:**
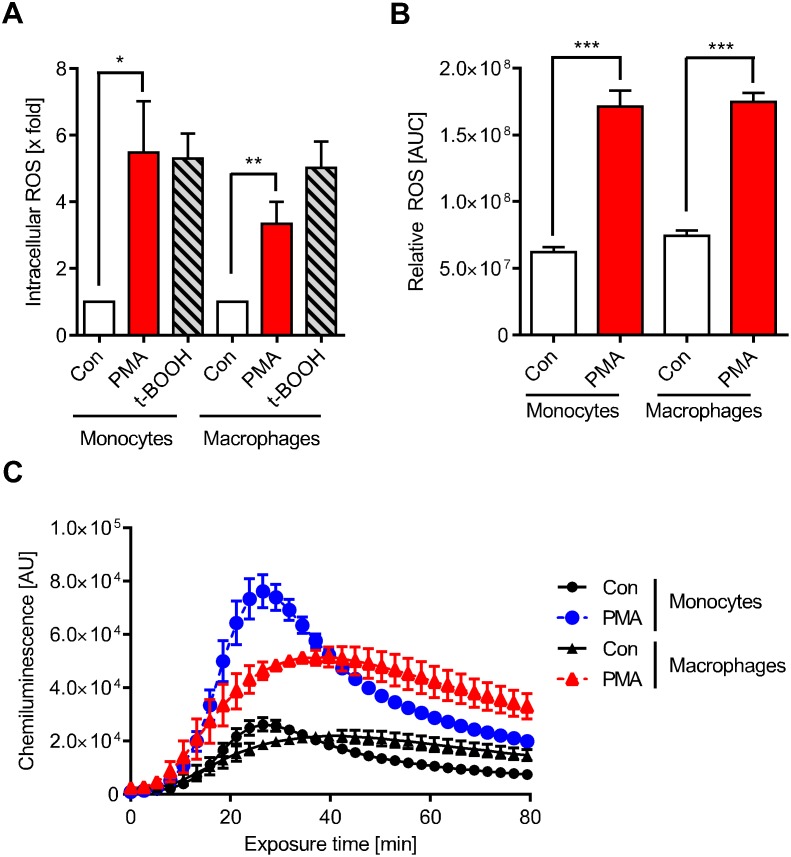
PMA-induced ROS formation. (A) Cells were stained with 10 μM CM-H2DCFDA immediately before treatment with PMA or t-BOOH for 30 min. PMA triggered intracellular ROS production similar to the positive control t-BOOH. (B) Extracellular ROS produced by monocytes and macrophages following treatment with PMA was measured via chemiluminescence with luminol plus HRP and then quantified. (C) The extracellular ROS production of monocytes and macrophages measured over time showed different kinetics. PMA was added to the cells at zero time and chemiluminescence was measured thereafter as described. Data are the mean of at least three independent experiments ± SEM, 1-way ANOVA, Dunnett's Multiple Comparison Test, *p < 0.05, **p < 0.01, ***p < 0.001.

PMA, an activator of protein kinase C (PKC) and downstream of NADPH oxidase (NOX) [17], is known to activate monocytes and macrophages to respond to a ROS burst [5–7]. Therefore, we set out to determine whether PMA induces ROS under our experimental conditions in monocytes and macrophages. As shown in Fig 2A, PMA triggered significant intracellular ROS production in monocytes and macrophages, which was similar to what we observed when cells were treated with the oxidative agent *t*-BOOH. ROS was also detected outside of the cell in the incubation medium (Fig 2B), indicating that NOX2 was activated, which produces reactive oxygen that is delivered to the external environment. Following activation by PMA, monocytes and macrophages were similarly effective in producing ROS (Fig 2B). However, the time course of ROS production is different, with a fast and strong response in monocytes that peaked at approximately 30 min compared to a weaker and more sustained response in macrophages (Fig 2C). Inhibition of NOX by an inhibitor, diphenyleneiodonium chloride (DPI), reduced the ROS production to control level (S3 and S4 Figs), confirming the notion that the extracellular ROS results from NOX activity. Pre-incubation of monocytes with N-acetylcysteine (5 mM) for 1 h also reduced the intracellular ROS level (S5 Fig). Overall, the data show that PMA activates the NADPH oxidase in our human fresh-blood derived monocyte/macrophage cell system.

**Fig 3:**
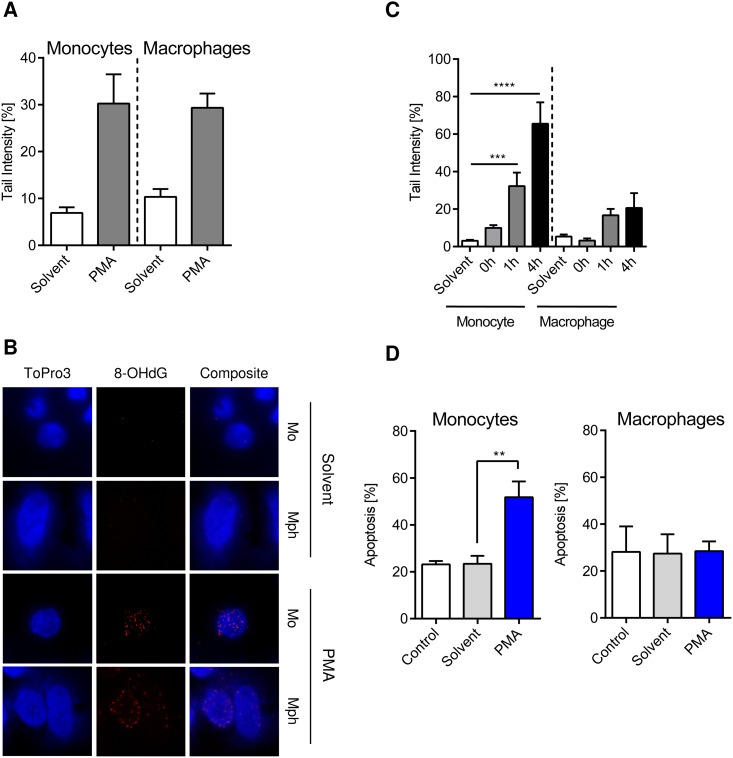
8-oxo-guanine (8OHdG) formation, DNA break induction and apoptosis in monocytes and macrophages following ROS burst resulting from PMA treatment. (A) The initial oxidative DNA damage was measured in monocytes and macrophages using an FPG-modified alkaline Comet assay. Cells were treated with PMA for 15 min and then incubated for additional 45 min in PMA free medium. Monocytes and macrophages showed similar levels of initial DNA damage. (B) In parallel, 8OHdG was detected via immunostaining. Both monocytes and macrophages displayed clear 8OHdG staining after PMA treatment compared to the solvent control. (C) DNA strand breaks were measured at different times after the ROS burst was induced by 15 min PMA treatment. Monocytes displayed increasing levels of strand breaks over time compared to macrophages. (D) Monocytes and macrophages were treated with PMA for 15 min and cell death was measured 48 h later. Monocytes displayed increased apoptosis compared to macrophages, which were resistant. Control, untreated cells; solvent, DMSO control (see material and methods). Data are the mean of at least three independent experiments ± SEM, 1-way ANOVA, Tukey’s Multiple Comparison Test, **p < 0.01, ***p < 0.001.

Next, we addressed the question whether the DNA of activated monocytes and macrophages is damaged through their own ROS burst. As revealed by the FPG-modified comet assay, which is very sensitive in detecting DNA strand breaks at 8-OHdG sites, PMA stimulation of monocytes and macrophages gives rise to 8-OHdG formation in their DNA. The 8-OHdG level was similar in monocytes and macrophages (Fig 3A), which was confirmed by immunodetection of 8-OHdG (Fig 3B). The data indicate that the DNA of monocytes and macrophages is a target of ROS they produce and that monocytes and macrophages display a similar level of initial oxidative DNA damage. Measuring DNA strand breaks as a function of time following the ROS burst using the alkaline comet assay, we observed in a 4 h post-incubation period a significant increase of strand breaks in monocytes, but not in macrophages (Fig 3C and S6 Fig). This can be explained on the basis of our previous published data showing that monocytes are able to perform the first steps of BER following ROS, including the incision step, but not the ligation step for strand breaks produced during BER. Macrophages, on the other hand, are able to execute the ligation step, reducing the amount of BER strand break intermediates [13]. Therefore, the repair defect in monocytes results in high amounts of DNA strand breaks several hours after the ROS burst. This is anticipated to result in higher cell death rates, which were actually observed in monocytes, but not macrophages, following ROS burst upon PMA stimulation (Fig 3D).

**Fig 4:**
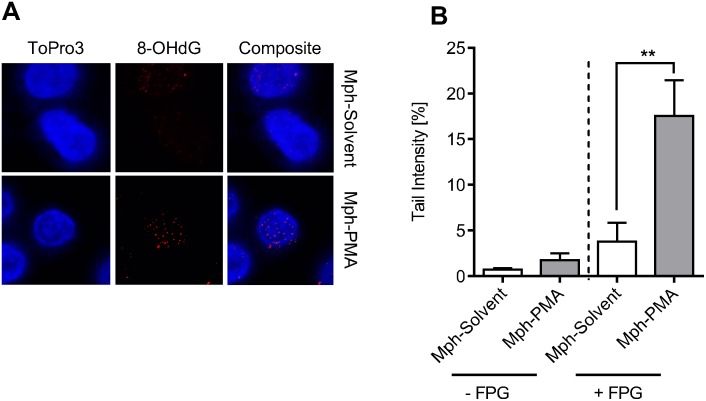
Monocytes in a co-culture setting with activated macrophages display oxidative DNA damage. (A) Monocytes were co-cultured with solvent-treated (Mph-Solvent) or PMA-activated (Mph-PMA) macrophages for 1 h before 8OHdG was detected. Monocytes exposed to activated macrophages displayed increased 8OHdG signals. (B) 1 h after co-culture with activated macrophages monocytes were analysed for oxidative DNA damage using the FPG-modified alkaline Comet assay. Monocytes exposed to PMA-activated macrophages (Mph-PMA) displayed increased DNA damage. Data are the mean of four independent experiments ± SEM, 1-way ANOVA, Tukey’s Multiple Comparison Test, **p < 0.01.

Having shown that monocytes and macrophages are similarly vulnerable to initial ROS-induced DNA damage and differ in their repair capacity, we assessed whether the ROS burst of macrophages is able to induce DNA damage in monocytes. Co-cultivation of PMA-activated macrophages with non-activated monocytes resulted in detectable 8-OHdG formation in monocytes, as revealed by immunostaining (Fig 4A). This was confirmed measuring the migration of DNA using the FPG-modified alkaline Comet assay. As shown in Fig 4B, in the presence of FPG, monocytes co-cultured with PMA-activated macrophages (Mph-PMA) displayed a significantly higher level of strand breaks than the solvent control (*i*.*e*. macrophages treated with DMSO; for representative Comet images see S7 Fig). Similar data were obtained by calculating the DNA damage on the basis of tail moment (S7 Fig). The data show that the level of extracellular ROS produced by activated macrophages is high enough to produce significant DNA damage in monocytes present in their vicinity.

**Fig 5:**
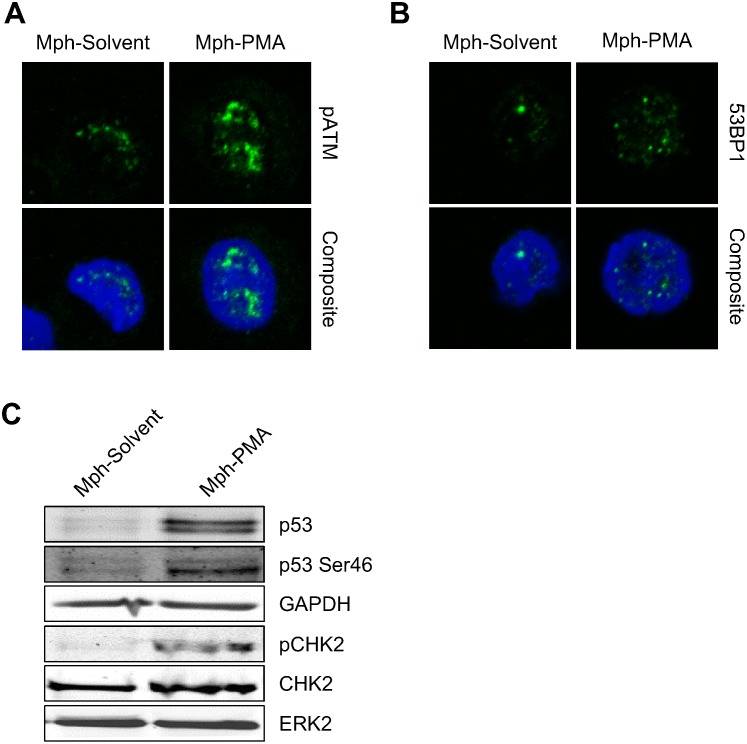
The DNA damage response was activated in monocytes co-cultured with activated macrophages for 24 h. (A) Monocytes displayed pATM immunostaining when they were co-cultured with activated macrophages (Mph-PMA), but not with non-activated macrophages (Mph-Solvent). (B) Monocytes showed increased 53BP1 foci formation after co-culture with activated macrophages. (C) There was stabilisation of p53 protein as well as phosphorylation of p53 at position Ser46 and phosphorylation of CHK2 in monocytes exposed to activated macrophages (Mph-PMA).

Does the ROS burst of activated macrophages result in activation of the DNA damage response (DDR) in monocytes? Co-cultivation of activated macrophages with monocytes gave rise to pATM and 53BP1 foci formation in monocytes (Fig 5A and 5B) and p53 accumulation, p53 phosphorylation at Ser46 and CHK2 phosphorylation (Fig 5C), indicating that the ROS produced by macrophages is sufficiently high to activate the DDR in monocytes.

**Fig 6:**
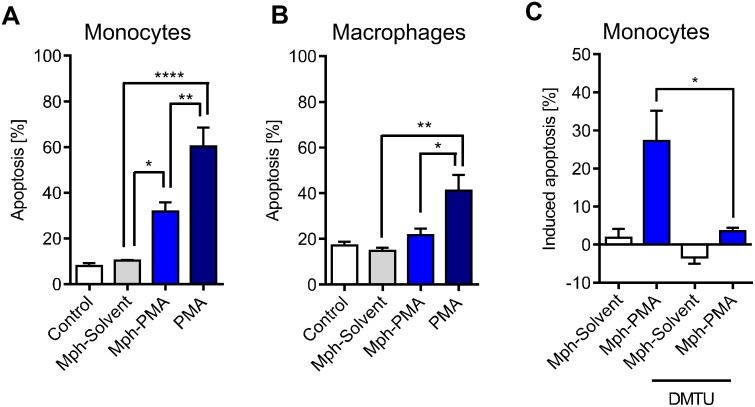
Apoptosis of monocytes and macrophages co-cultured with non-activated and activated macrophages. (A) Monocytes were co-cultured with un-treated (Control), solvent-treated (Mph-Solvent) or PMA-activated (Mph-PMA) macrophages and apoptosis was measured 48 h later. Monocytes and macrophages were also concomitantly exposed to PMA (PMA) and apoptosis was measured after 48 h. Exposure of both cell types to PMA exacerbated the killing effect in monocytes. (B) Macrophages themselves showed in the same experimental setting no toxicity after short-term treatment with PMA (Mph-PMA) and only a moderate increase in cell death after direct exposure to PMA (PMA). (C) Monocytes were co-cultured with solvent-treated or PMA-activated macrophages in the absence or presence of the ROS scavenger DMTU (10 mM). In the presence of DMTU, they showed significantly less cell death than monocytes co-cultured with macrophages in the absence of ROS scavenger. Data are the mean of at least four independent experiments ± SEM, 1-way ANOVA, Tukey’s Multiple Comparison Test, *p < 0.05, **p < 0.01, ***p < 0.001.

Next, we assessed whether ROS produced by activated macrophages is on a level that provokes a killing response (apoptosis) in monocytes present in their vicinity. To this end, we performed co-culture experiments using macrophages, which were pre-labelled with CFSE. Following co-culture, we separated monocytes and macrophages by flow cytometry and determined apoptosis by Annexin V staining. Monocytes co-cultured with PMA-activated macrophages (Mph-PMA) displayed a significantly higher level of apoptosis than monocytes co-cultured with non-treated macrophages (control) or DMSO-treated macrophages (solvent) (Fig 6A; for representative plots see S2 Fig). As expected, co-cultivation of monocytes with macrophages, both exposed to PMA for a period of 48 h, exacerbated the killing effect in monocytes (Fig 6A). Under the same conditions, PMA-pulse activation of macrophages did not lead to apoptosis in the macrophages themselves (Fig 6B), indicating that they were protected from cell death resulting from their own ROS burst, confirming data shown above (Fig 3D). Following long-term (48 h) PMA treatment, significant levels of cell death was also observed in macrophages, which was lower, however, than in monocytes (Fig 6B). The killing effect caused by co-cultured activated macrophages (we termed it *killing in trans*) is dependent on ROS since the addition of the radical scavenger DMTU to the medium in the co-culture setting completely abolished apoptosis in monocytes (Fig 6C).

**Fig 7:**
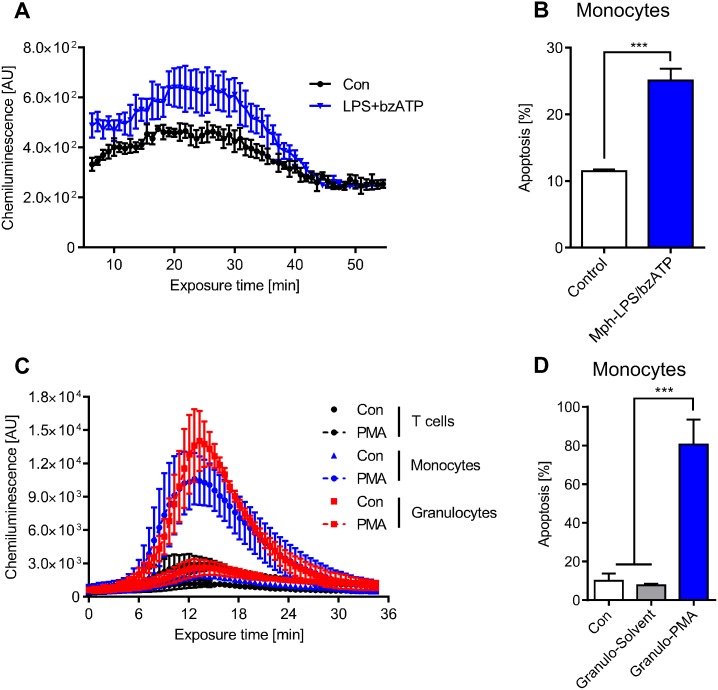
LPS/bzATP-activated macrophages and PMA-activated granulocytes produce ROS that kills monocytes in co-culture. (A) ROS production in LPS/bzATP-activated macrophages. LPS/bzATP was added to the cells at zero time. (B) Apoptosis of monocytes co-cultured for 24 h with LPS/bzATP-activated macrophages. (C) Extracellular ROS produced by granulocytes as a function of time following addition of PMA to the medium. The kinetics were similar to ROS produced by activated monocytes. T cells are shown for comparison; they do not produce ROS. (D) Monocytes were co-cultured with solvent-treated (Granulo-Solvent) or PMA-activated granulocytes (Granulo-PMA) and apoptosis was measured in monocytes after 48 h. Data are the mean of four independent experiments ± SEM, 1-way ANOVA, Tukey’s Multiple Comparison Test, ***p < 0.001.

Treatment of macrophages with LPS together with bzATP was reported to induce a ROS burst [18], which was confirmed by our data shown in Fig 7A. Co-cultivation of monocytes with LPS/bzATP activated macrophages resulted in significant apoptosis in monocytes (Fig 7B).

Besides macrophages, neutrophilic granulocytes also produce ROS following stimulation. The ROS level is even higher than in monocytes (Fig 7C). Therefore, we expected *killing in trans* if monocytes were co-cultured with activated granulocytes. This was indeed the case; control and solvent (DMSO) treated granulocytes were not able to kill monocytes in their vicinity while PMA-stimulated granulocytes did (Fig 7D). The killing effect was dramatic as nearly 80% of monocytes in the co-culture underwent apoptosis. It was comparable to long-term PMA treatment of monocytes itself (S7 Fig). Collectively, the data demonstrate that monocytes are sensitive to ROS produced by activated macrophages and neutrophilic granulocytes, displaying a high amount of DNA damage and undergoing significant cell death by apoptosis.

**Fig 8:**
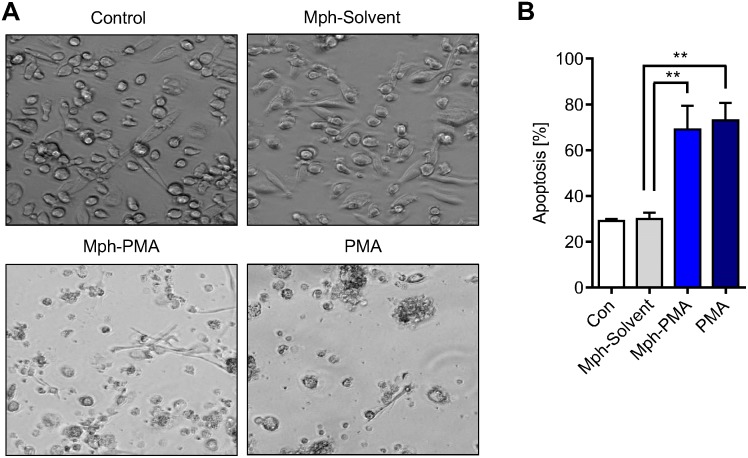
Maturation of monocytes by GM-CSF after co-cultivation with PMA-activated macrophages. (A) Representative images of monocytes cultivated after co-culture with macrophages in the presence of GM-CSF. Monocytes were co-cultured with untreated (Control), solvent-treated (Mph-Solvent) or PMA-stimulated macrophages (Mph-PMA). Thereafter monocytes were separated from macrophages, re-seeded and differentiated into macrophages by adding GM-CSF. On day 6 the generated macrophages were analysed. (B) Analysis of the cell population on day 6 showed a high level of apoptosis (Annexin V+) in cells previously co-cultured with PMA-stimulated macrophages (Mph-PMA) or directly treated with PMA for 24 h (PMA). Data are the mean of at least three independent experiments ± SEM, 1-way ANOVA, Bonferroni's Multiple Comparison Test, **p < 0.01, ***p < 0.001.

In the presence of GM-CSF, monocytes differentiate into macrophages. Is this differentiation process impaired if they are cultured in the presence of ROS producing macrophages? Addressing this question, we co-cultured monocytes and PMA activated macrophages for 24 h. Thereafter, monocytes were harvested, re-seeded and cultured in the presence of GM-CSF for six days to achieve their differentiation into macrophages. In the controls (monocytes co-cultured with macrophages not treated or treated with solvent only), GM-CSF-treated monocytes were able to fully differentiate into macrophages. However, when monocytes were co-cultured with PMA treated macrophages (Mph-PMA) the majority of monocytes died during their maturation period (Fig 8A). The cells remaining on the dish after six days of maturation were harvested and subjected to Annexin V staining, which revealed a high yield of Annexin V positive cells in the population (Fig 8B). Control experiments with PMA treatment for 24 h of both monocytes and macrophages displayed the same high killing response in monocytes cultivated in the presence of GM-CSF (Fig 8A and 8B). The data indicate that a fraction of monocytes, which is not immediately killed by the ROS burst originating from macrophages, undergoes death during the GM-CSF triggered maturation period and is therefore unable to differentiate into macrophages.

## Discussion

The experiments reported here were performed to gain support for the hypothesis that the DNA repair defect in monocytes reported previously [12,13,16] has biological consequences in terms of selective monocyte killing in a ROS enriched environment. Following the release from the bone marrow, monocytes circulate in the blood stream for a short time (1–3 days [19]) and, following passage through the blood vessel endothel, migrate into tissues where they differentiate into macrophages and DCs [20,21]. These cell types represent key nodes in the immune response following infection, chronic inflammation and cancer. DCs present foreign antigens on their surface, thus activating cytotoxic T cells. Macrophages on the other hand respond following activation with a ROS burst, thereby killing pathogens that are later engulfed by macrophage phagocytosis [1–4]. Also monocytes respond with ROS production following activation, and the same is true for neutrophilic granulocytes (as shown in Figs 2 and 7). It is generally accepted that the most abundant and relevant genotoxin in the inflamed area (following infection or acute and chronic tissue damage including cancer) is ROS, generated by monocytes, macrophage, and neutrophils.

While monocytes and neutrophils are short lived cells, macrophages reside in the tissue for longer times [1]. Therefore, it is conceivable that ROS produced especially by macrophages has the potential to damage normal cells during the inflammatory response. Many side effects of excessive ROS production are ascribed to chronic inflammation in tissues. Thus, accumulation of DNA damage in inflamed areas was reported to result in tissue degeneration, necrosis, presumably also fibrosis and, as late effects, cell transformation and cancer [22,23]. With this scenario in mind, it is reasonable to posit that the amount of ROS producing cells, notably monocytes and macrophages, present in an inflamed environment are subject to regulation. How could this be achieved? A reasonable mechanism would require a biological sensor that detects ROS and signals if a given (critical) ROS level is achieved, thus regulating the amount of monocytes and, therefore also indirectly, the amount of monocyte derived macrophages. A sensitive biological sensor for ROS is nuclear DNA, in which oxidised products such as 8-oxo-guanine and DNA single-strand breaks are formed upon oxidative stress. That this happens is shown in this study, demonstrating the formation of 8-oxo-guanine and DNA strand breaks in monocytes and macrophages that were stimulated to produce ROS. We also show here that co-cultivation of monocytes with activated ROS-producing macrophages produces a significant amount of 8-oxo-guanine and strand breaks in monocytes (DNA damage *in trans*), indicating that the ROS level in the environment of activated macrophages is high enough to cause significant DNA damage in monocytes in their vicinity.

Oxidised DNA lesions are quickly repaired by BER, and single- and double-strand break repair pathways are involved in this response [24,25]. Therefore, cells tolerate a given amount of ROS until the repair system becomes saturated. In DNA repair defective cells, however, small amounts of ROS already have an impact on the viability of cells, which was shown for many established mutant cell lines [26–28] and also for monocytes, which are impaired in BER and DSB repair [13]. Due to incomplete BER and the persistence of DNA repair intermediates, monocytes activate the DDR and the downstream apoptotic pathway following exogenous genotoxic insults while DNA repair competent macrophages are resistant and not activate the DDR [16]. In order to kill monocytes, a particular level of exogenous ROS is required [13]. In view of this, we wondered whether the ROS burst produced by activated monocytes and macrophages is sufficiently high to elicit a genotoxic effect in these cells. Therefore, we extended these studies to ROS produced by monocytes and macrophages and show that activated monocytes have a high susceptibility to die as a result of their own ROS burst (death by suicide) compared to macrophages. Most importantly, the ROS burst of macrophages was strong enough to induce in monocytes a significant level of DNA damage, activating the DDR by stabilizing p53, activating p53Ser46, which is a pro-apoptotic marker [29] and CHK2 and finally provoking apoptotic death of monocytes. This effect, termed *killing in trans*, was completely abolished by a ROS scavenger added to the co-culture. It was also observed upon co-cultivation of monocytes with activated ROS-producing neutrophils.

It is reasonable to posit that the death of monocytes resulting from their own ROS burst (see S8 Fig) and *in trans* from ROS produced by macrophages (and neutrophils) has an impact on the amount of macrophages, as a fraction of tissue macrophages originates from monocytes. Also, the maturation of monocytes into macrophages might be hampered. Thus, it is conceivable that this scenario contributes to a reduction in the overall ROS level in a given inflamed area. In this context, it is important to note that monocytes are non-replicating cells in which DSBs do not result from replication blockage. They are likely the result of overlapping BER patches, which requires a sufficient amount of DNA base lesions. Therefore, monocytes are able to tolerate a low amount of ROS and start to die if this level exceeds a given threshold [13]. Thus, the DNA of the monocytes may be considered to be a perfect sensor for ROS, triggering death if monocytes are no longer able to tolerate the DNA damage.

**Fig 9:**
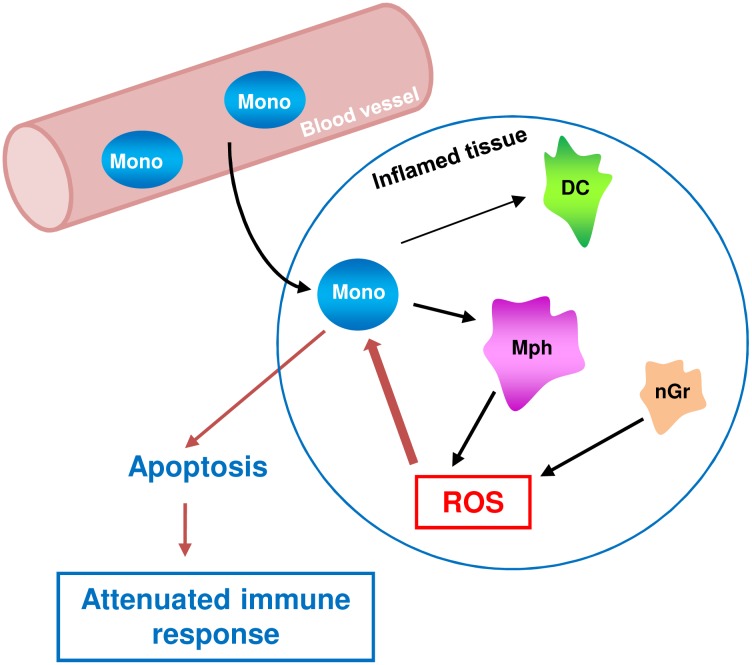
Model of regulation of the immune response by killing of monocytes *in trans*. Since monocytes are not only precursors of macrophages but also dendritic cells (DC), their selective killing may also have an impact on the amount of DCs and DC-mediated responses during infection and inflammation.

In summary, we provide evidence that activated macrophages are able to induce DNA damage and killing of monocytes *in trans*. Cell death of monocytes results from oxidative DNA damage and BER intermediates, which are known to trigger the DNA damage response and apoptotic pathways in monocytes [16]. Importantly, while monocytes are killed, macrophages sustain the same ROS level and survive due to their efficient BER and DSB repair system [12,13]. Monocytes are also the precursor of dendritic cells (DCs), which are protected against ROS similar to macrophages [13]. Therefore, the death of monocytes in a ROS-enriched environment will also have an impact on the amount of DCs and DCs-mediated responses. Our data support the hypothesis that the impaired DNA repair capacity of monocytes and their high vulnerability to ROS (compared to macrophages) is a mechanism involved in regulating the innate immune response (Fig 9). In this model, the DNA in monocytes is a physiological sensor for ROS with amplification of the damage by incomplete DNA repair, transforming the ROS-induced primary adducts into toxic lesions. The non-repaired DNA lesions may also have an impact on the proper function of monocytes. According to this hypothesis, the ultimate consequence of the defective DNA repair is death of the monocyte in a ROS-enriched inflammatory environment, in part executed by a suicide mechanism, restricting the amount of monocytes and monocyte-derived macrophages. Consequently, the amount of ROS, which is a potential harm to normal cells, will be limited and the healthy tissue protected.

## Supporting Information

S1 Fig Purity of granulocytes isolated from buffy coats. Granulocytes isolated from four different buffy coats (Granulocyte 1 to 4) were almost exclusively CD15 positive. Granulocytes stained with CD15 were also shown in the PBMC fraction isolated by Ficoll gradient centrifugation.
(TIF)

S2 Fig Exemplary gating for apoptotic monocytes co-cultured with macrophages. Monocytes were gated for CD14+ and CFSE exclusion. Then monocytes were gated for Annexin V positivity.
(TIF)

S3 Fig Inhibition of ROS production by NADPH oxidase inhibitor DPI—Time course. The extracellular ROS production of monocytes was measured over time. ROS production was completely abolished with 100 μM NADPH oxidase inhibitor diphenyleneiodonium chloride (DPI). ROS production steeply increased and peaked at ~30 min. In macrophages the extracellular ROS production was weaker but persisted compared to monocytes. Data are the mean of at least three independent experiments.
(TIF)

S4 Fig Inhibition of ROS production by NADPH oxidase inhibitor DPI—Quantification. Quantification of the extracellular ROS generated by monocytes. ROS production was completely abolished with 100 μM NADPH oxidase inhibitor DPI. Quantification of the extracellular ROS generated by macrophages after PMA treatment. Inhibitor DPI reduced ROS production below control level. Data are the mean of at least three independent experiments ± SD, 1-way ANOVA, Dunnett's Multiple Comparison Test, ***p < 0.001
(TIF)

S5 Fig Intracellular ROS formation after PMA treatment reduced by ROS scavenger. Monocytes were treated with 5 mM N-acetyl cysteine (NAC) for 1 h prior treatment. Then, cells were stained with 10 μM CM-H2DCFDA immediately before treatment with 1, 10, 100 or 1000 ng/ml PMA. Mean fluorescence was measured via flow cytometry. Data was normalised to the untreated dye control. The ROS formation was at highest level at ~100 ng/ml PMA. ROS scavenger NAC reduced the intracellular ROS burden. Data are the mean of at least three independent experiments ± SD, 1-way ANOVA, Dunnett's Multiple Comparison Test, *p < 0.05, **p < 0.01
(TIF)

S6 Fig DNA single strand break formation in monocytes and macrophages after ROS burst. Cells were pulse-treated with PMA for 15 min and then incubated for up to 4 h. The monocytes displayed increased DNA strand breaks over time. Macrophages were resistant. Data are the mean of at least three independent experiments ± SEM, 1-way ANOVA, Tukey’s Multiple Comparison Test, **p < 0.01, ***p < 0.001
(TIF)

S7 Fig DNA single strand break formation in co-cultured monocytes. Monocytes co-cultured with PMA-activated macrophages for 45 min displayed DNA SSB in the FPG-modified alkaline Comet assay (Mph-PMA). Representative images of Comet tails show higher fragmentation of the DNA. Data are the mean of four independent experiments ± SD, 1-way ANOVA, Dunnett's Multiple Comparison Test, *p < 0.05, **p < 0.01, ***p < 0.001
(TIF)

S8 Fig Apoptosis in monocytes after short and long-term PMA treatment. Monocytes displayed increased apoptosis after PMA-pulse treatment (Mono-PMA). The effect was exacerbated when PMA treatment lasted for 48 h. Data are the mean of four independent experiments ± SD, 1-way ANOVA, Tukey’s Multiple Comparison Test, *p < 0.05, ****p < 0.001
(TIF)
