# Supplementary figures and images for: Death of Monocytes through Oxidative Burst of Macrophages and Neutrophils: Killing in Trans
Source: PLoS One. 2017 Jan 18;12(1):e0170347. doi: 10.1371/journal.pone.0170347 (PMC5242493; doi:10.1371/journal.pone.0170347)

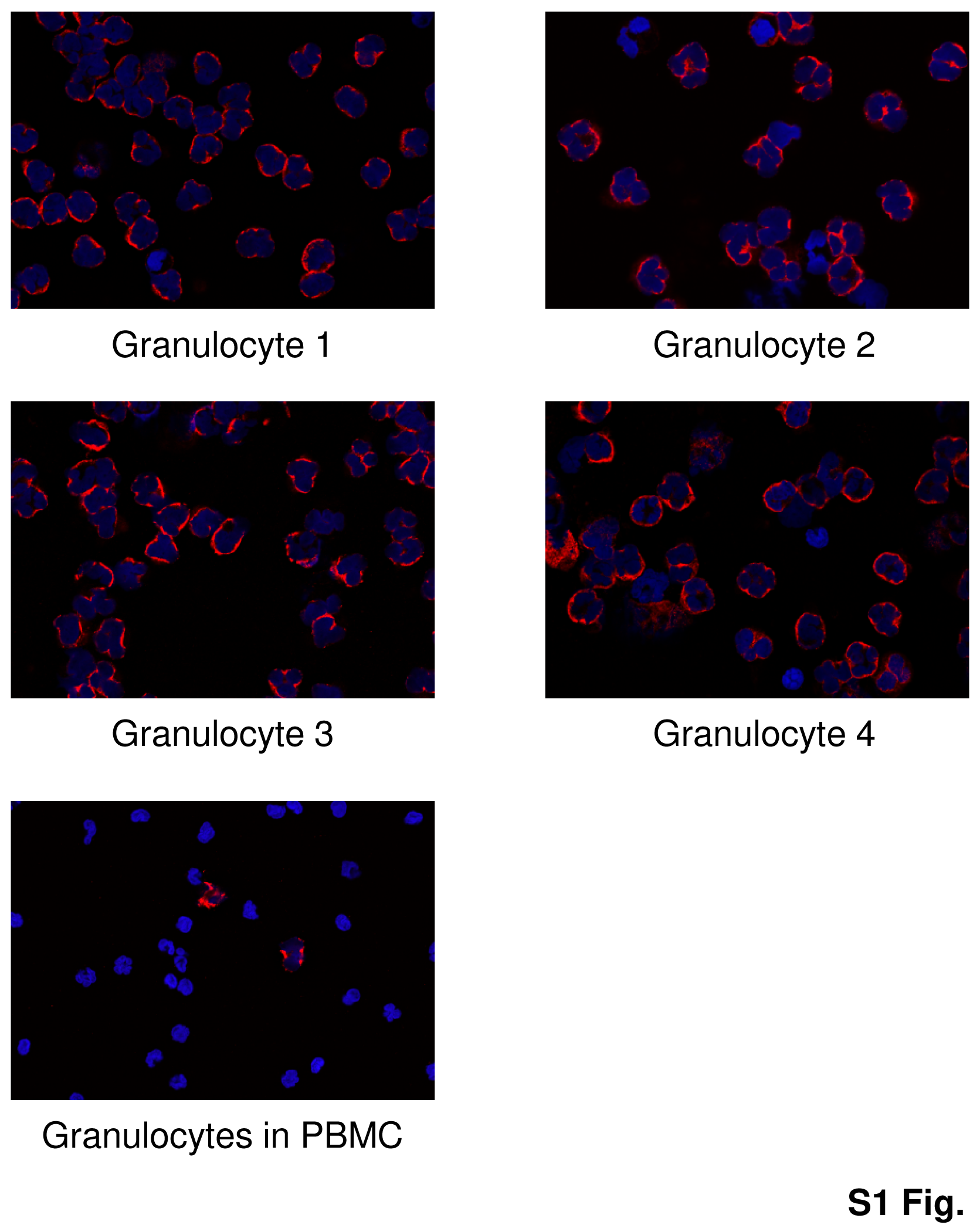

Supplement: S1 Fig — Granulocytes isolated from four different buffy coats (Granulocyte 1 to 4) were almost exclusively CD15 positive. Granulocytes stained with CD15 were also shown in the PBMC fraction isolated by Ficoll gradient centrifugation. (TIF) [file pone.0170347.s001.tif]

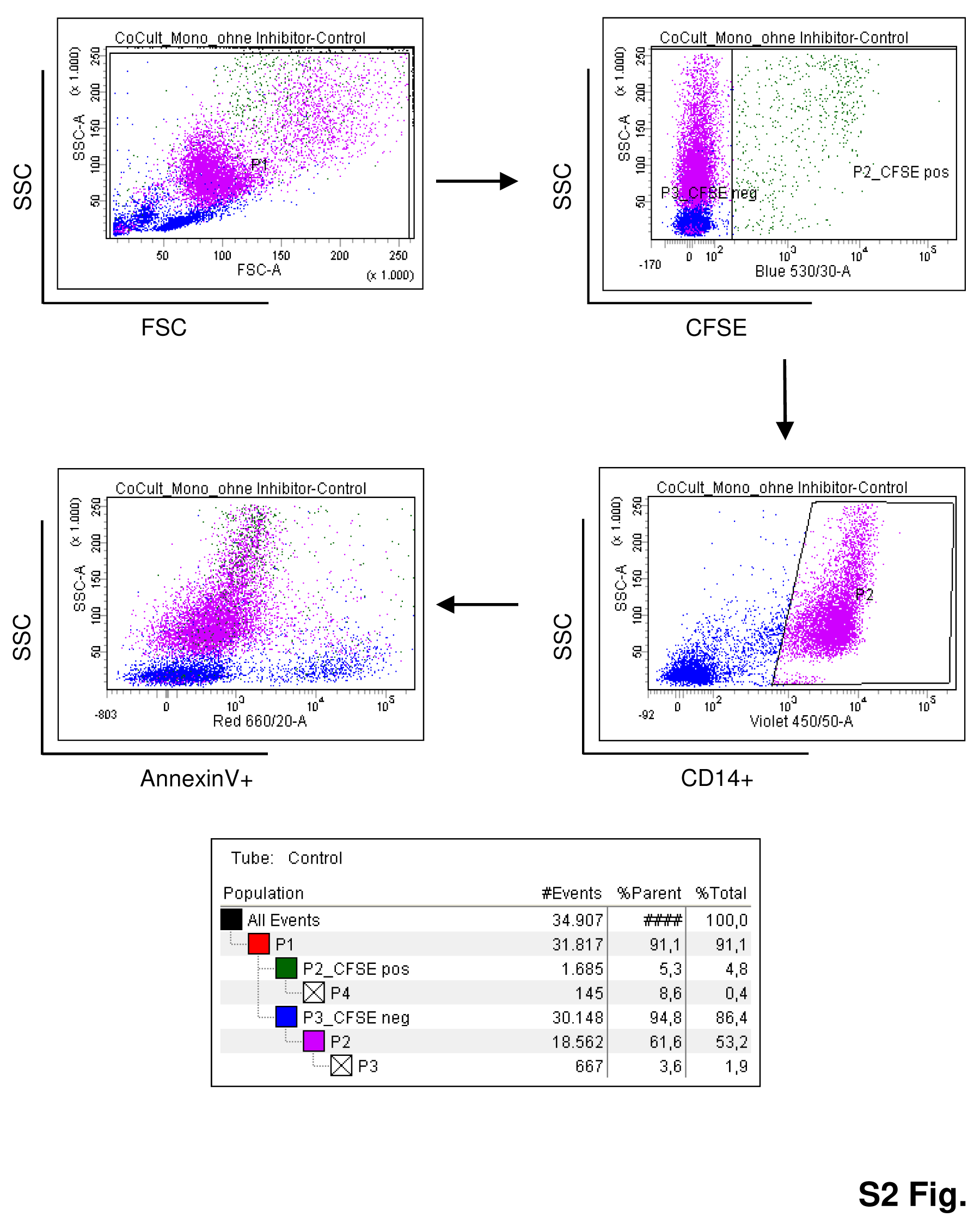

Supplement: S2 Fig — Monocytes were gated for CD14+ and CFSE exclusion. Then monocytes were gated for Annexin V positivity. (TIF) [file pone.0170347.s002.tif]

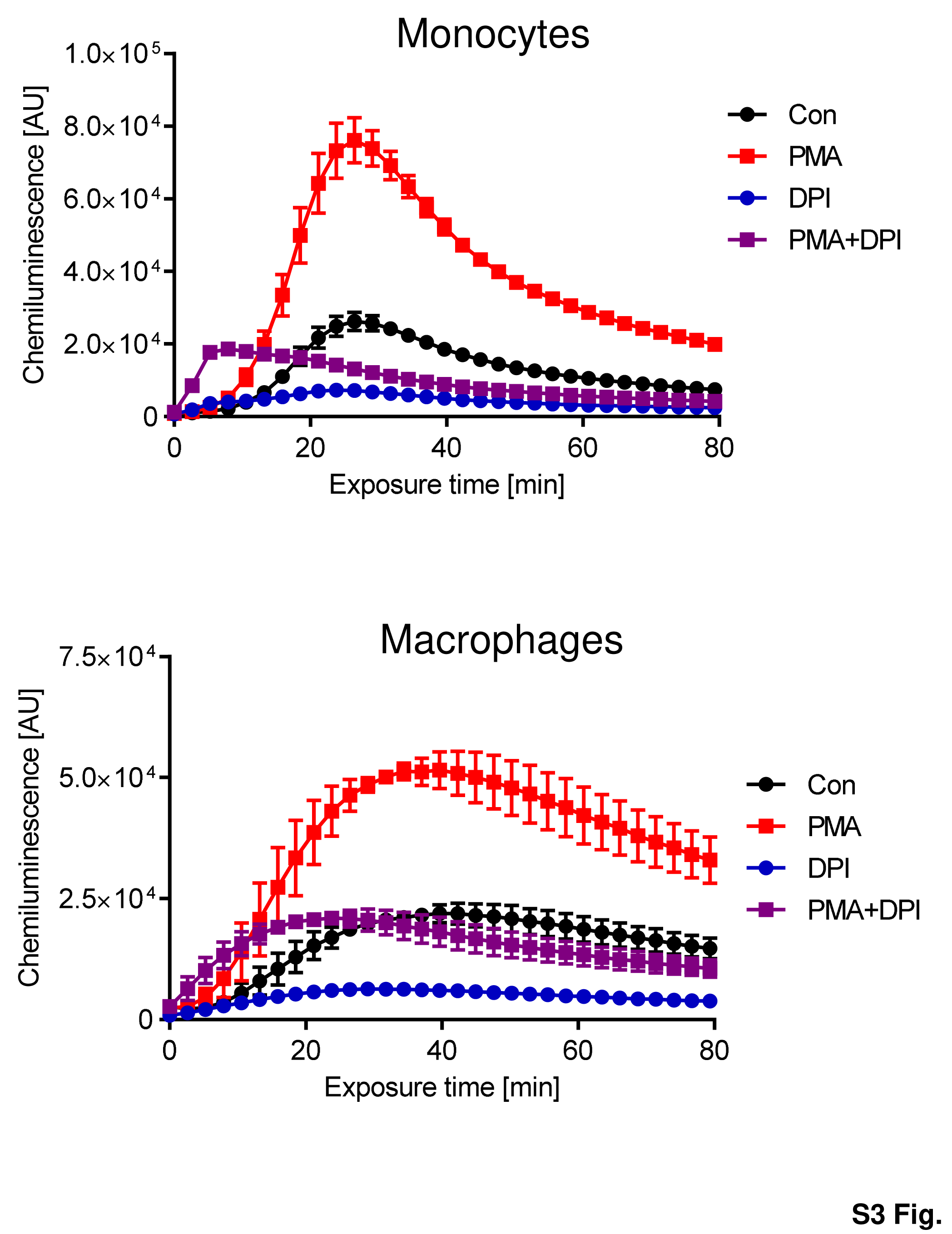

Supplement: S3 Fig — The extracellular ROS production of monocytes was measured over time. ROS production was completely abolished with 100 μM NADPH oxidase inhibitor diphenyleneiodonium chloride (DPI). ROS production steeply increased and peaked at ~30 min. In macrophages the extracellular ROS production was weaker but persisted compared to monocytes. Data are the mean of at least three independent experiments. (TIF) [file pone.0170347.s003.tif]

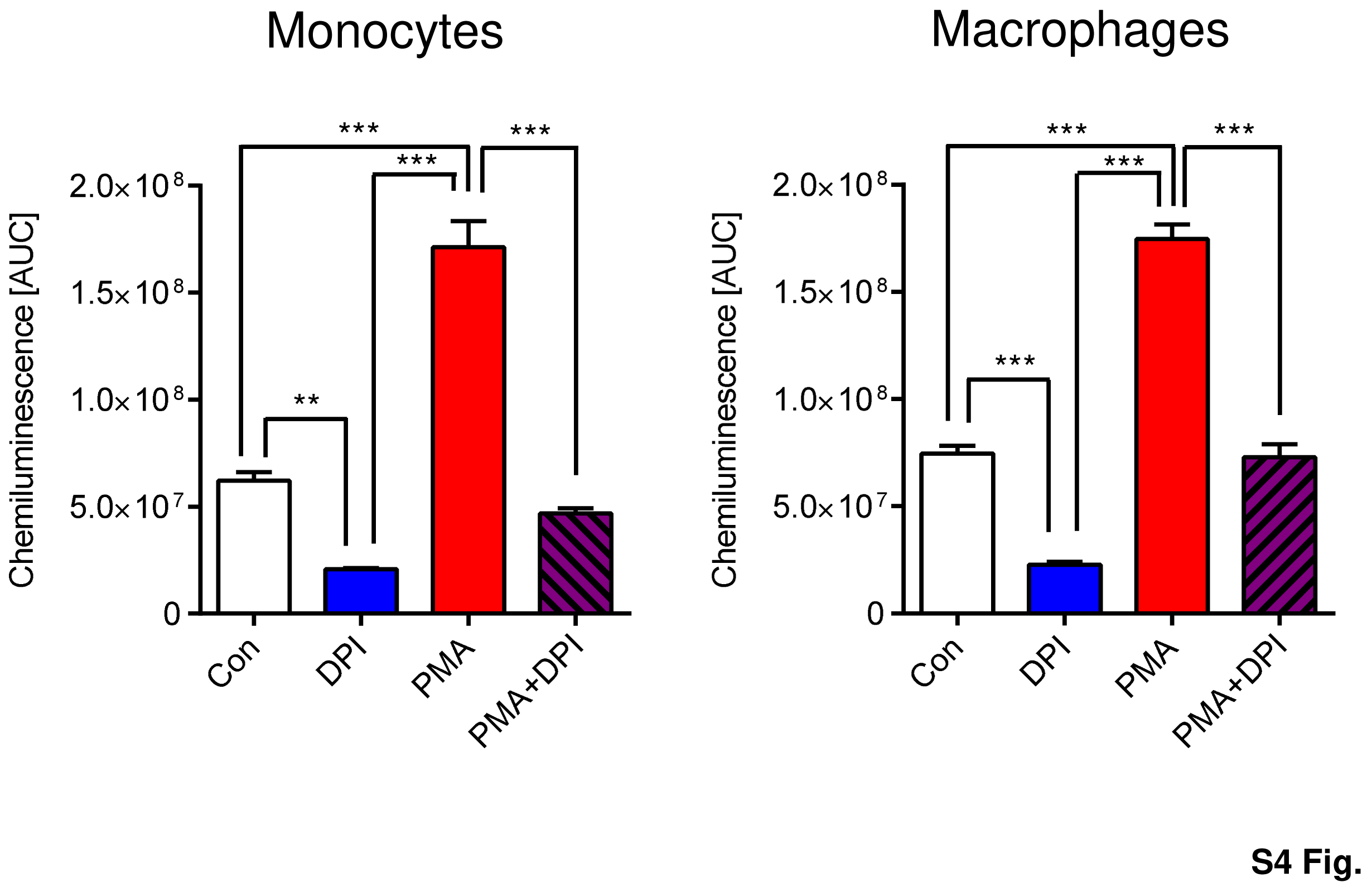

Supplement: S4 Fig — Quantification of the extracellular ROS generated by monocytes. ROS production was completely abolished with 100 μM NADPH oxidase inhibitor DPI. Quantification of the extracellular ROS generated by macrophages after PMA treatment. Inhibitor DPI reduced ROS production below control level. Data are the mean of at least three independent experiments ± SD, 1-way ANOVA, Dunnett's Multiple Comparison Test, ***p < 0.001 (TIF) [file pone.0170347.s004.tif]

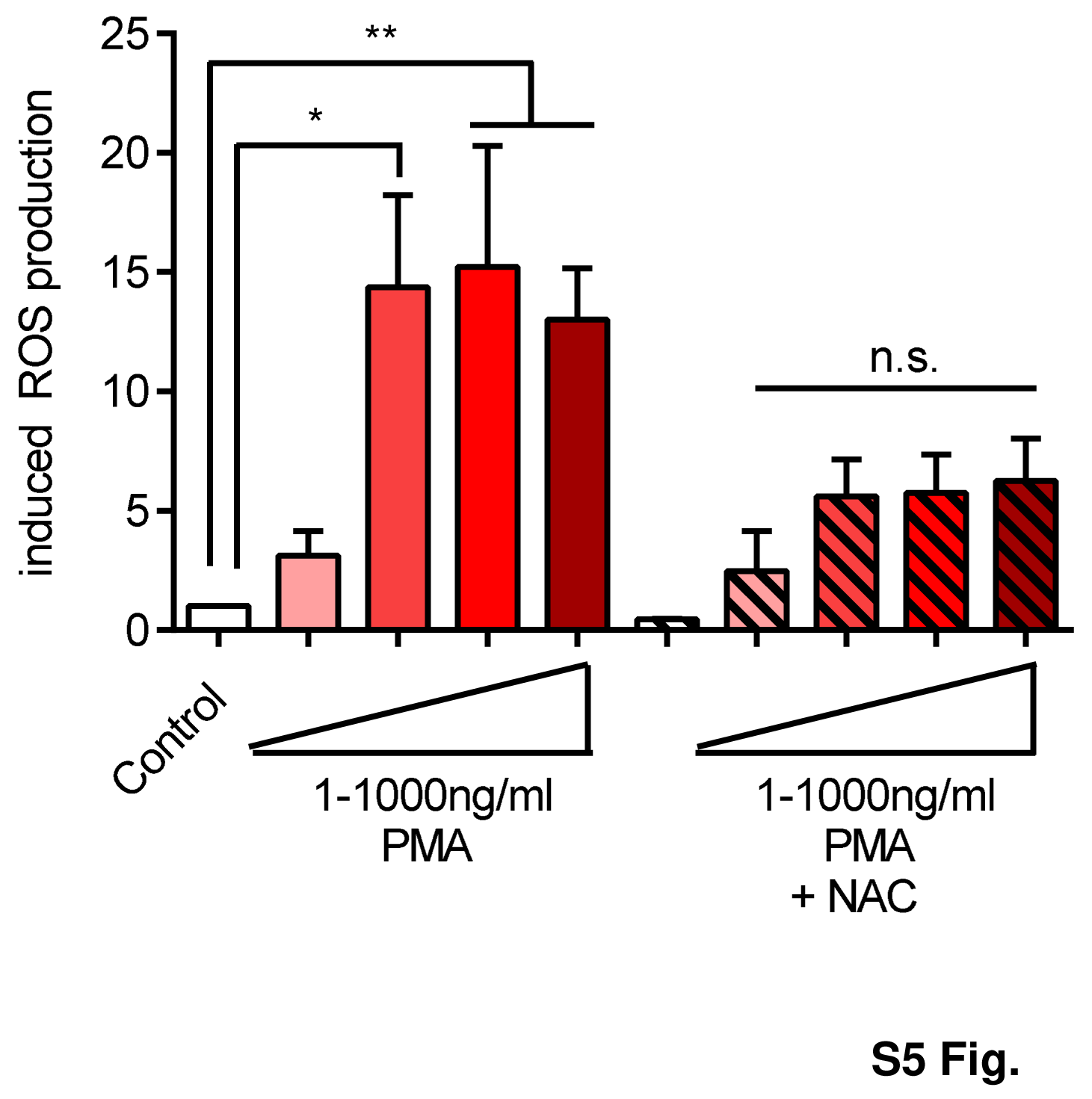

Supplement: S5 Fig — Monocytes were treated with 5 mM N-acetyl cysteine (NAC) for 1 h prior treatment. Then, cells were stained with 10 μM CM-H2DCFDA immediately before treatment with 1, 10, 100 or 1000 ng/ml PMA. Mean fluorescence was measured via flow cytometry. Data was normalised to the untreated dye control. The ROS formation was at highest level at ~100 ng/ml PMA. ROS scavenger NAC reduced the intracellular ROS burden. Data are the mean of at least three independent experiments ± SD, 1-way ANOVA, Dunnett's Multiple Comparison Test, *p < 0.05, **p < 0.01 (TIF) [file pone.0170347.s005.tif]

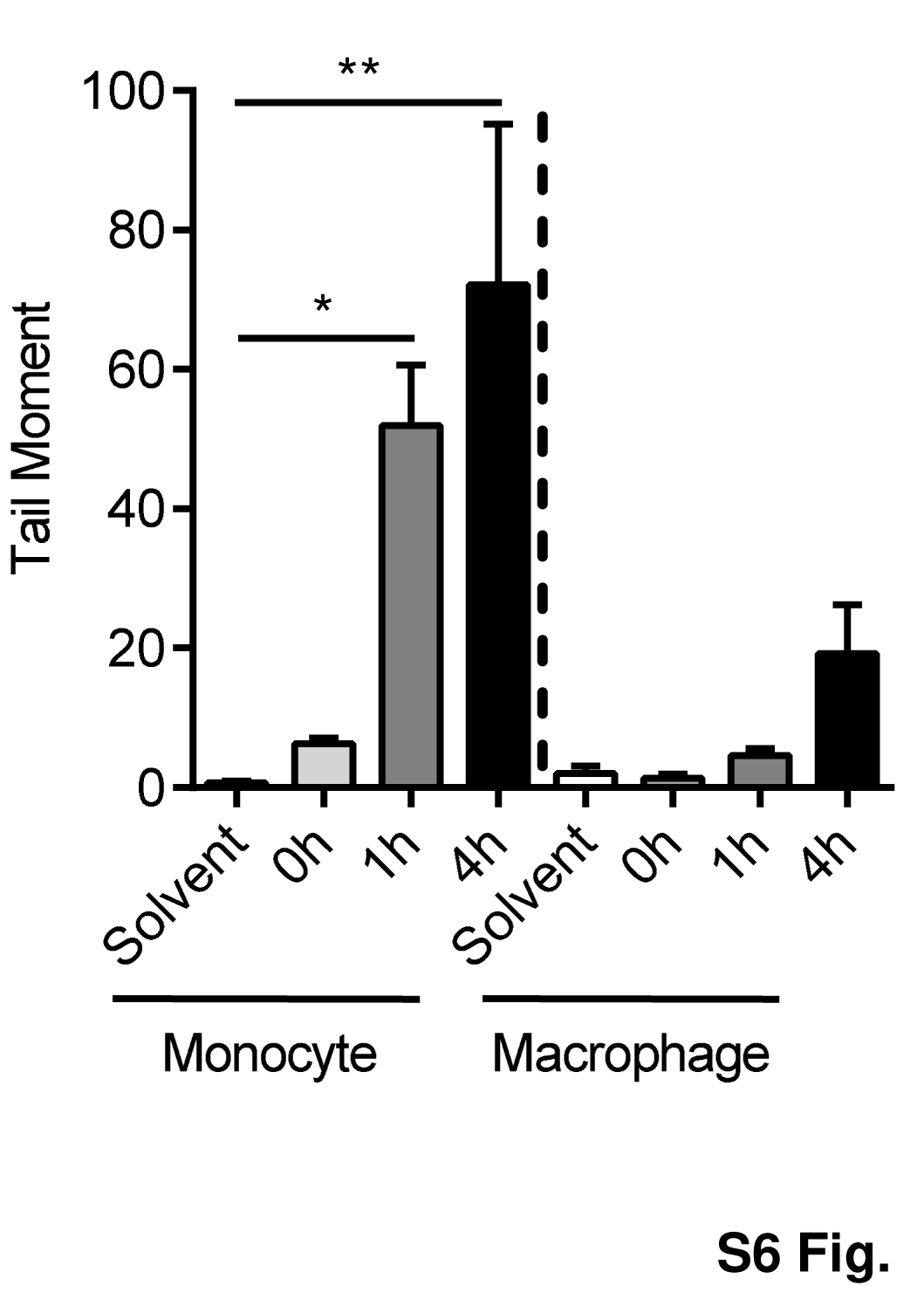

Supplement: S6 Fig — Cells were pulse-treated with PMA for 15 min and then incubated for up to 4 h. The monocytes displayed increased DNA strand breaks over time. Macrophages were resistant. Data are the mean of at least three independent experiments ± SEM, 1-way ANOVA, Tukey’s Multiple Comparison Test, **p < 0.01, ***p < 0.001 (TIF) [file pone.0170347.s006.tif]

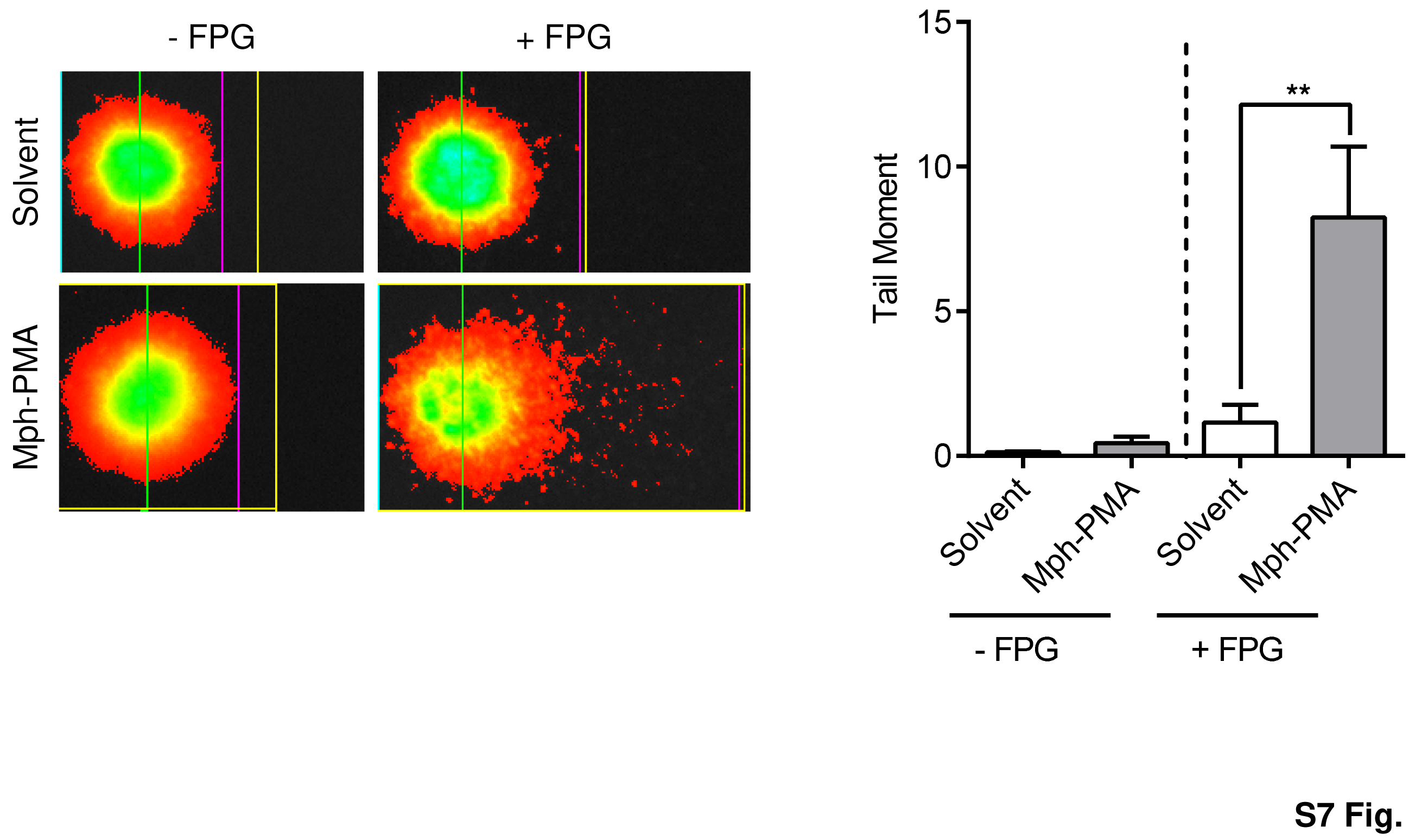

Supplement: S7 Fig — Monocytes co-cultured with PMA-activated macrophages for 45 min displayed DNA SSB in the FPG-modified alkaline Comet assay (Mph-PMA). Representative images of Comet tails show higher fragmentation of the DNA. Data are the mean of four independent experiments ± SD, 1-way ANOVA, Dunnett's Multiple Comparison Test, *p < 0.05, **p < 0.01, ***p < 0.001 (TIF) [file pone.0170347.s007.tif]

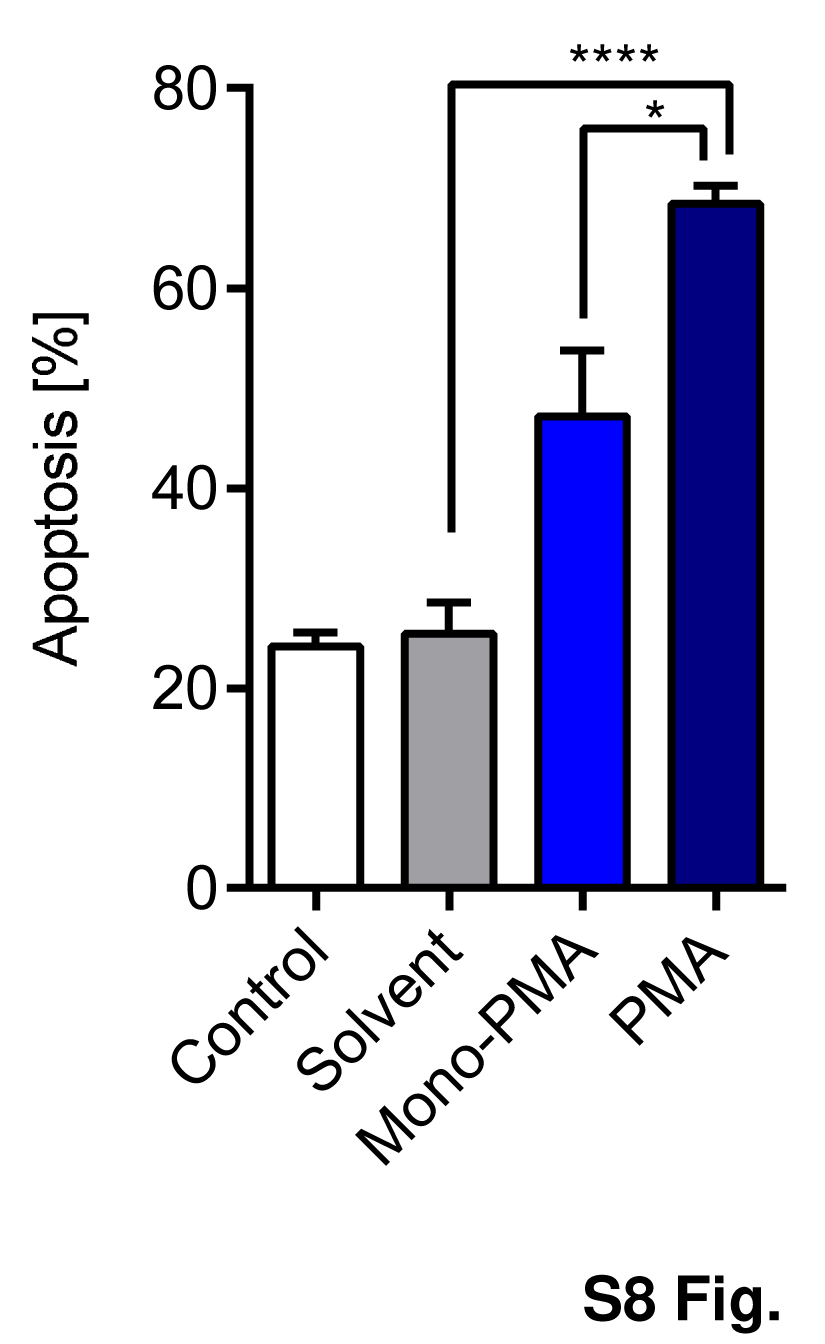

Supplement: S8 Fig — Monocytes displayed increased apoptosis after PMA-pulse treatment (Mono-PMA). The effect was exacerbated when PMA treatment lasted for 48 h. Data are the mean of four independent experiments ± SD, 1-way ANOVA, Tukey’s Multiple Comparison Test, *p < 0.05, ****p < 0.001 (TIF) [file pone.0170347.s008.tif]
